# Supplementation of Alpha-lipoic acid-loaded nanoliposomes in semen extender improves freezability of buffalo spermatozoa

**DOI:** 10.1038/s41598-022-26960-y

**Published:** 2022-12-28

**Authors:** Mahmoud A. E. Hassan, Wael A. Khalil, Sameh A. Abdelnour, Reham Mokhtar Aman

**Affiliations:** 1 Animal Production Research Institute, Agriculture Research Centre, Ministry of Agriculture, Dokki, Giza, 12619 Egypt; 2 Department of Animal Production, Faculty of Agriculture, Mansoura University, Mansoura, 35516 Egypt; 3 Department of Animal Production, Faculty of Agriculture, Zagazig University, Zagazig, 44511 Egypt; 4 Department of Pharmaceutics, Faculty of Pharmacy, Mansoura University, Mansoura, Dakahlia, 35516 Egypt

**Keywords:** Biotechnology, Developmental biology, Zoology

## Abstract

This research was designed to explore the protective effect of alpha-lipoic acid–loaded nanoliposomes (ALAN) during cryopreservation of buffalo sperm. Buffalo semen was cryopreserved in a tris-citrate egg yolk extender without any supplement (ALAN0, control group) or with ALAN at levels of 25, 50, 75 or 150 µg (ALAN25, ALAN50, ALAN75 and ALAN150, respectively). The ALAN had a size of 171.80 nm and a negative zeta potential (− 43.40 mV). The progressive motility, vitality and membrane integrity significantly improved in all ALAN groups (except ALAN25 for membrane integrity). ALAN150 group exhibited the best values of progressive sperm motility, vitality and membrane integrity after thawing at 37 °C for 30 s or incubated for 2 h at 37 °C and 5% CO_2_ compared with those in other groups. Both ALAN75 and ALAN150 groups significantly improved the TAC, GR and catalase, while lipid peroxidation and early apoptotic spermatozoa significantly decreased in ALAN150 group followed by ALAN75 group. Collectively, the adding ALAN to buffalo semen freezing extender plays a substantial shielding function against cryodamage by preserving the sperm functional parameters.

## Introduction

Sperm cryopreservation (SPC) in domestic animals is a significant reproductive biotechnology, extensively applied to preserve genetic resources, enhance male fertility and control diseases. With SPC, it was confirmed that the fruitful application of reproductive technologies such as artificial insemination (AI) and in vitro fertilization (IVF) to boost the rapid genetic plausible of domestic animals^1,2^. Although cryopreservation system offers many benefits as previously described, it causes impairment to various sperm cell parts including protein, DNA, and membrane lipids which may decrease sperm functionality and ultimately ability to fertilization^2,3^. In our previous works, sperm structural and functionality was changed by cryopreservation method in different animals^4–6^. Furthermore, structural fluctuations of sperm plasma membranes, principally lipid–protein composition ratio, kind of extender, nature of cryoprotectants, cooling–thawing rates, and periodic differences, restrict the prosperity of cryopreservation prevalence among all species^7,8^. Studies reported that buffalo sperm membrane has a high level of saturated and polyunsaturated fatty acids contents, which make them vulnerable to the lethal impairments caused by unwarranted reactive oxygen species (ROS) release during cryopreservation^7,9,10^. Naturally, spermatozoa have an enzymatic antioxidant defense system^11^. The imbalance between the antioxidant defense and oxidative stress generations (including ROS) in spermatozoa during the cryopreservation leading to damages sperm membrane, and DNA injury as well as induce reduction in sperm quality and subsequently constrain the ability to fertilize^4,5,12^. However, the natural antioxidant defense system in spermatozoa is ineligible to resist the ROS triggered impairments. Posteriorly, supplementation of antioxidant in semen extender is a robust policy to retrograde the unfavorable impacts of ROS^9^.

**Figure 1:**
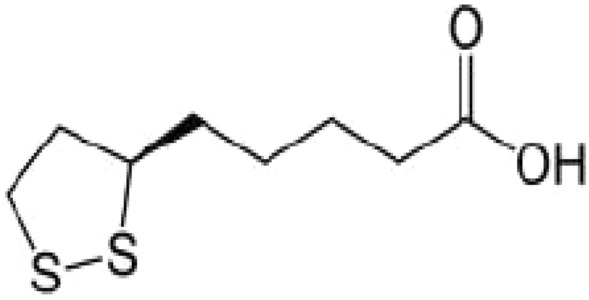
Chemical structure of Alpha Lipoic Acid.

Alpha-lipoic acid (ALA) (Fig. 1) is a non-vitamin naturally detecting in the mitochondria, which playing a critical role in the mitochondria function^10^. ALA is an organo‐sulphur molecule (di‐thiol) derivative from octanoic acid and represents as an essential cofactor for the enzyme system in the body^13^. Moreover, ALA is unity of the utmost robust biological antioxidants. This powerful antioxidant can effortlessly sneak various cells, tissues, and even organelles such as mitochondria as chief apparatus driving ROS synthesis^14,15^.

Accordingly, supplementation of ALA (0.5 mmol/L) in freezing extenders can enhance buffalo sperm cryosurvival, motility, structure and function, due to reduced oxidative stress and boosted energy production^16^. Studies have confirmed that adding ALA in the freezing extender exhibited several positive impacts on post-thawing semen variables such as sperm motility, viability, acrosome, DNA and membrane integrities in goats^3,15^, boar^12^, and ram^17^. Adding ALA (0.02 and 0.5 mM) to semen samples acquired from sterility men shows a substantial defensive function alongside cryodamage by sustain the sperm structure and functional^7^. Still, a limitation of usage ALA, in terms of medical, feed additives, cryoprotective agent, and cosmetic uses, was detected because of its poor water solubility in aqueous solutions^13,18^. Nanotechnology has recently been received more attention by scientists due to its widely uses in several fields such animal science, agricultural, pharmaceutical, engineering, and cosmetic industries^18,19^. Nanoliposomes (NLPs) are spherical vesicles from phospholipids and have a hydrophilic head and a hydrophobic tail^19^. Consequently, NLPs have been chosen as delivery systems. The use of alpha-lipoic acid–loaded nanoliposomes (ALAN) as a new active ingredients delivery system could enhance the solubility, stability and effectiveness of ALA. In the current work, the manipulation of ALA encapsulating it in NLPs formulation was executed to obviate the dilemmas of ALA. We theorized that enrichment of semen extender with ALAN before cryopreservation would perfect the semen quality of buffalo, by enhancing sperm functionality, reduce the apoptotic sperm and boosting the antioxidant capacity. Hence, the intent of the existing research is to evaluate the impacts of ALAN on cryopreservation damages of buffalo sperm, assessing post-thawing sperm quality and ultrastructure changes.

## Material and methods

ALA was kindly provided as a gift sample from Future Pharmaceutical Industries Company (Cairo, Egypt). Soybean lecithin (SL) was purchased from Carlo Erba Reagents S.A.S. (Chaussée du Vexin, France). Chloroform and methanol (HPLC grade, Fischer) were acquired from Cornell lab (Cairo, Egypt). This study was approved by the Scientific Research Ethics Committee of Mansoura University in accordance with Animal Research Reporting of in Vivo Experiments (ARRIVE) guidelines. Moreover, all experimental procedures and research protocols were performed in accordance with the guidelines and regulations laid down and duly approved by Scientific Research Ethics Committee of Mansoura University.

### Preparation of ALA-loaded and ALA-free nanoliposomes

The conventional thin-film hydration technique was followed in the preparation of ALAN, as earlier depicted, with slight modifications^20^. Briefly, accurately weighed quantities of SL and ALA, 50 and 10 mg, respectively, were dissolved in 10 mL of the organic solvent (a blend of methanol and chloroform in a ratio of 1:2 v/v). In a round-bottom flask, the organic solvent mixture was evaporated into a transparent thin film under vacuum by rotary evaporator (Heidolph LABOROTA 4000, Serial No. 030002422, Germany) at 60 °C (Heidolph Heizbad WB, Serial No. 020004125, Germany), while rotating at 70 revolutions per minute (rpm) till complete evaporation of the organic solvent. Such dried thin lipid film was hydrated with deionized water (DW; 10 mL) for 20 min at 60 °C and 120 rpm to have a crude nanoliposomal suspension. Subsequently, the suspension was dispersed by sonication for 30 min via an ultrasonic bath (Sonix USA, SS101H230) and further homogenized by utilizing an ultrasonic probe (Serial No. 2013020605, Model CV 334) attached to a homogenizer (Sonics Vibra-cell™, Model VC 505, Sonic & Materials, INC., USA) in an ice bath under the following conditions: (Amplitude: 60%, Timer: 3 min, Pulser: 1 s ON/ 1 s OFF) to yield one-phase NLPs. Finally, the obtained NLPs dispersions were stored at 4 °C until use. For preparation of the corresponding ALA-free nanoliposomes (NLPs_free_), without ALA in the organic solvent mixture, the same procedure was followed.

### Physicochemical characterization of ALAN

#### Determination of ALA entrapment efficiency percent (EE %)

Efficiency of ALA entrapment as ratio (EE %) was indirectly assessed. Succinctly, free ALA was separated from the prepared ALAN using centrifugal concentration at 6000 rpm for 30 min (ACCULAB Cooling centrifuge, CE16-4X100RD, USA) using Amicon^®^, 4 mL and 10 KDa cutoff units, Ultra-4 Centrifugal Filter Units (Bioscience Research Reagents, Merck Co., California, USA). Then, unentrapped ALA quantity in the ultrafiltrate was assessed spectrophotometrically, against the ultrafiltrate of NLPs_free_ as a blank, at 242 nm (JENWAY 6850 ultraviolet/visible (UV–VIS) double beam spectrophotometer, UK).

The EE % was calculated according to the following Eq. (1):

1 $$EE \%=\frac{{ALA}_{total\; amount}-{ALA}_{unentrapped\; amount}}{{ALA}_{total\; amount}}\times 100$$

Furthermore, both NLPs_free_ as well as ALAN were washed with DW, resuspended in DW, and then freeze-dried (SIM FD8-8T, SIM international, USA). Ultimately, the freeze-dried samples were preserved at 4 °C for further characterization.

#### Determination of ALAN vesicular size

ALAN average vesicular size (Z-average) besides polydispersity index (PDI) were evaluated using Zetasizer Nano ZS analyzer (Malvern Instruments, Malvern, UK) and evaluated by dynamic light scattering (DLS) technique. Such aforesaid measurements, in triplicates after adequate dilution with DW, were carried out for the freshly prepared formula at 25 °C.

#### Determination of ALAN surface charge

ALAN surface charge, being expressed as zeta potential (ZP) which detects the vesicles' electrophoretic mobility in an electric field, was estimated in triplicates, utilizing the same apparatus besides the same dilution in DW, as mentioned in the aforementioned Z-average measurement.

#### Transmission Electron Microscopy (TEM)

Morphology of the freshly attended ALAN was conceived on transmission electron microscope (TEM) (JEOL JEM-2100, Tokyo, Japan). After adequate dilution with DW besides sonication, the carbon-coated copper grid was evenly covered with nanoliposomal dispersion, air-dried at room temperature and finally inspected directly via TEM without any staining at 160 kV. Digital Micrograph (V 2.11.1404.0) and Soft Imaging Spectator software were utilized to complete the image capture, analysis process, besides particle sizing.

#### Fourier transform-infrared spectroscopy (FT-IR)

The infrared spectra of pure ALA, SL, their physical mixture corresponding to the prepared formula (accurately weighed quantities of 10 mg ALA and 50 mg SL) besides the freeze-dried NLPs_free_ as well as ALAN were recorded through the FT-IR Spectrophotometer (Madison Instruments, Middleton, WI, USA). Thereafter, the samples were homogeneously and individually combined with potassium bromide, flattened into discs and scanned (thirty-two scans) over a wavenumber range of 4000–500 cm^−1^.

#### Differential scanning calorimetry (DSC)

Differential scanning calorimetry (DSC) can be considered as a complementary technique that provide beneficial information concerning the thermal behavior evaluation of the loaded NLPs. To appraise such thermic properties, ALA, SL, their physical mixture corresponding to the prepared formula besides the freeze-dried NLPs_free,_ and ALAN samples were traced using differential scanning calorimetry (DSC-60A Plus, Shimadzu Corporation, Japan). Each sample (4 mg), in a hermetically sealed aluminum pan, was heated from 30 to 200 °C at a heating rate of 10 °C/min in a saturated inert environment (20 mL/min of dry nitrogen). Indium (melting point of 156.6 °C and purity of 99.99%), was used as a reference to calibrate DSC runs. Finally, the respective heat flow curves were recorded, displayed, and analyzed using a Shimadzu TA-60 Series data processor.

#### X-ray diffraction analysis (XRD)

X-ray diffraction (XRD) technique, which allows structural investigations of materials' crystallinity, is a non-destructive, environmentally, and user-friendly analytical tool which makes use of X-rays to penetrate powders, crystals and liquids deeply and quickly. To inspect crystallinity changes throughout NLPs formularization process, XRD patterns of ALA, SL, their physical mixture corresponding to the prepared formula besides the freeze-dried NLPs_free,_ and ALAN samples were recorded utilizing a Diano X-ray diffractometer (USA) equipped with Co-Kα radiation. To accomplish such analysis process, a scanning range from 3° to 50° at 2θ angle, 9 mA current as well as 45 kV voltage were applied.

#### Comparative stability studies of ALA-loaded NLPs

For comparative stability studies performance, freshly prepared ALAN dispersions were stored, for three months in sealed glass bottles, at accelerated storage conditions (25 ± 1 °C, 60 ± 5% RH) as well as long-term storage conditions (4 ± 1 °C), without any stirring or agitation^21^. The NLPs were evaluated in what concerns judicious forefingers for their kinetic stability namely; physical appearance, Z-average, PDI, ZP besides average drug retention (%). Such aforementioned measurements were carried out initially (at production day as previously detailed above), and over the period of storage.

### Animals and semen collection

A twenty-five semen ejaculates were picked up from five water buffalo bulls (*Bubalus bubalis*) aged 3–5 years maintained at the breeding center at Mahalet Mussa close to Sakha, Kafr El-Sheikh Governorate, Egypt, under standard management and feeding conditions.

Semen samples were collected weekly for a period of 5 weeks. Semen was collected routinely by artificial vagina technique (pre-warmed at 42 °C). After semen collection, semen was transferred directly into the laboratory and two qualified researchers evaluated every ejaculate for sperm motility using a phase contrast microscope (100X). Then, ejaculates having minimum progressive motility (75%), volume (4 mL), abnormality (15%), livability (80%), and sperm cell concentration (500 X10^6^/mL) were selected, pooled, and utilized in freezing trials.

#### Freezing procedures

Semen extender was prepared under standard construction procedures in International Livestock Management Training Center (ILMTC) conferring to Khalil et al.^6^ with some modifications. Freezing extender was provided by dissolving 3.028 g of Tris, 1.675 g of citric acid, 1.25 g of fructose, 6.0 mL of glycerol, 20 mL of egg yolk, and antibiotics (100 IU/mL of penicillin, and 100 µg/mL of streptomycin) in double distilled water and the volume was made up to 100 mL. After preparation, semen samples were diluted with the previous freezing extender with the extension rate 1 semen: 10 extenders. The diluted semen was transferred into five test tubes and kept at 37 °C in a water bath. Semen extended in five tubes were supplemented with different doses of alpha-lipoic acid–loaded nanoliposomes (ALAN) at 0, 25, 50, 75 and 150 µg/mL, designated as groups; ALAN0, ALAN25, ALAN50, ALAN75, and ALAN150, respectively. After that, the extended semen samples were preserved in a cooling chamber for 4 h at 5 °C as an equilibration period, then automatically occupied in 0.5 mL French straws (IVM technologies, L0 Aigle, France), then positioned at 4 cm above liquid nitrogen for 10 min then frozen in liquid nitrogen (− 196 °C) as depicted by Salisbury et al.^22^. Samples (5 replicates in each treatment) were estimated in equilibrated buffalo bull semen (at 5 °C for 4 h), after thawing in water bath (at 37 °C for 30 s) and incubation after thawing (at 37 °C and 5% CO_2_ for 2 h).

#### Semen evaluation

##### Progressive Motility

The ratio of progressive sperm motility in individually semen sample was assessed via phase contrast microscope (Leica DM 500, Schweiz) provided with a hot stage set at 37 °C. Concisely, aliquots of diluted sperm (10 µl diluted semen) were placed on pre-warmed glass slides and covered with a glass cover slip^4,23^. Examination progressive motility was detected in 4–5 various fields of microscope for individually semen sample. The average of the consecutive considerations of motility were recorded as the ending motility value^24^.

##### Viability and abnormalities

Spermatozoa viability was assessed via the one-step eosin-nigrosin staining method^25^. A 10 µL from each sample was made on a glass slide and then it was stained by eosin (1.67%) and nigrosine (10%) stain. At least 200 sperm cells in each sample were assessed under light microscope (400X; Leica DM 500, Schweiz). Sperm cells which were unstained or with white coloration were considered as living, while those with red coloration in the head district were reflected as dead. Additionally, in the same field the number of sperm cells bearing head and tail morphological abnormalities were also documented as beforehand identified^23^.

##### Membrane Integrity

To assess the membrane integrity of spermatozoa, hypo-osmotic swelling (HOS) test as depicted by Khalil et al.^6^. HOS solution (osmolality = 75 mOsmol/L) is consisted of solution (3.67 g/L sodium citrate and 6.75 g/L fructose). In a brief, fifty micro liters of diluted semen were mixed with HOS solution (500 μL) and was incubated at 37 °C for 30 min in water bath. After incubation, 10 µl of the mixture was placed on a micro-scope slide and mounted with a coverslip. A two hundred of sperm were evaluated for their swelling competence in HOS solution. Spermatozoa with coiled tail and swollen were measured to have an intact plasma membrane^26^.

##### Acrosome integrity

Semen samples (50 µL) in each experimental groups were fixed in 500 µL of a 1% formaldehyde citrate in 2.9% (w/v) tri-sodium citrate dihydrate. A total of 200 spermatozoa in each sample were counted (Phase-contrast microscope, X 1000) for two categories of acrosome assessments^23^.

##### Apoptosis-like changes

The apoptotic alterations of spermatozoa were performed using the annexin V staining protocol with minor amendments, as previously reported by^27^. Concisely, semen extended (1 mL) was added in a sterilized tube (5 mL) containing binding buffer (2 mL) and the suspension was mixed well. Then, sperm suspension (100µL) was transferred into a sterilize tube (5 mL), and the same volume of annexin V (5 µL; FITC label) and PI (5 µL; PE label) were directly added, then the suspension was incubated in dark conditions for 15 min at room temperature. Then, the samples were suspended in a 200 µL of binding buffer. The spermatozoa were ready for Flowcytometric evaluation (within 5 min). The Flowcytometric examination were implemented using the Accuri C6 Cytometer (BD Biosciences, San Jose, CA) attached with Accuri C6 software (Becton Dickinson) for analysis and acquisition^28^. The analyze of annexin V-FITC binding by flow cytometry (Ex = 488 nm; Em = 350 nm) using FITC signal detector (FL1) and PI staining by the phycoerythrin emission signal detector (FL2). Sperm were classified according to^27^ in the following four patterns: (1) live sperm (A−/PI−), (2) early apoptotic sperm (A+/PI−), viable spermatozoa were labeled with Annexin-V but not with PI (3) dead, late apoptotic or early necrotic sperm (A+/P+), spermatozoa bound both PI and Annexin-V that having scratched permeable membranes and (4) dead, necrotic sperm (A−/P +), spermatozoa was bound with PI but not Annexin-V and have entirely lost sperm membrane. The number of replicates in each treatment for this test were three times.

##### Ultrastructure changes

Semen samples were administered for studying the ultrastructure changes in sperm using TEM as designated by^4,6^ with some amendments. In a brief, semen samples (200–300 µL) were exposed to centrifugation and suspended in a fixative solution (glutaraldehyde in PBS 2.5%) at 4 °C for two hours. After that, samples were washed and fixed with osmium tetroxide (1%) for 90 min at room temperature. The fixed samples were dehydrated in an ethanol with different gradient, then washed and treated with propylene oxide and ultimately embedded in Epon 812 (Fluka Chemie, Switzerland) and ultrathin-sectioned (60–70 nm). Ultrathin segments were detected via a TEM (JEOL-JEM 2100) at 80 kV. In each treated group and the control, around 200–300 sperm in each one was examined for studying sperm ultrastructure changes.

### Assessment of antioxidant, oxidative markers and enzymatic activities in extender of post-thawed buffalo bull semen

Post-thawed semen samples (five replicates in each treatment) were exposed to centrifugation for 10 min at 6000 rpm, then seminal plasma was detached and preserved at − 20 °C. The levels of TAC (total antioxidant capacity), GR (Glutathione reductase), and MDA (Malondialdehyde) in seminal plasma were measured using commercially available reagent kits (Biodiagnostic Company, Giza, Egypt). The procedures were accomplished by subsequent the manufacturer’s directives. The enzymatic activities of AST (aspartate aminotransferase), ALT (Alanine aminotransferase), ALP (alkaline phosphatase) and TAP (total acid phosphatase) in seminal plasma after thawing were assessed using special commercial kits providing by Biodiagnostic Company (Giza, Egypt).

### Statistical analysis

Collected data were edited in Microsoft Excel (Microsoft Corporation, Redmond, WA, USA), then subjected to A Shapiro–Wilk test for checking to normality. Data were subjected to analysis of variance using one way ANOVA (Proc ANOVA; SAS Institute Inc.,2012) with the level of significance set at α = 0.05. The following statistical model was applied for analysis of all measurements

$${\text{Yij}} =\upmu + {\text{TRTi}} + {\text{eij}}.$$

where Yij = Observations, μ = Overall mean, TRT = effect of the ALAN concentrations (i, 1–5), eij = random error. Results were expressed as means ± SE.

Tukeys’ test was used to perform pairwise comparisons between means in case of a significant effect was detected. Statistical significance between means was set at p-value less than 0.05. Figures were fitted by the GraphPad Prism software 9.0 (GraphPad, USA).

## Results

### Physicochemical Characterization of ALAN

**Figure 2:**
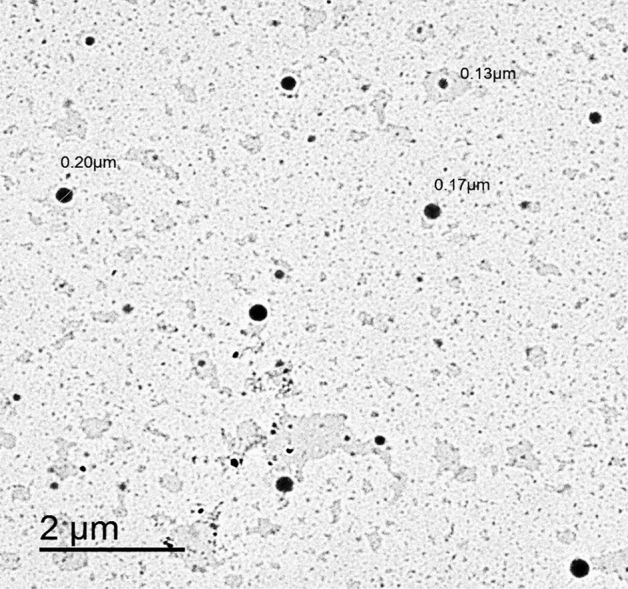
The image of synthesized alpha lipoic acid nanoparticles loaded liposomes (ALAN) characterized by transmission electron microscope (type JEOL-JEM 2100).

EE % was found to be 96.46% ± 0.12 indicating that almost all of ALA was entrapped. The values of Z-average, PDI, and ZP of ALAN were found to be 171.80 ± 2.60 nm, 0.264 ± 0.01, and -43.40 ± 3.35 mV, respectively. Imaging of the freshly prepared delicate NLPs dispersions were obtained using an ultra-high resolution electron beam of the JEOL TEM microscope. Such images depicted the spherical morphology of the ALAN with little or no aggregation observed (Fig. 2).

**Figure 3:**
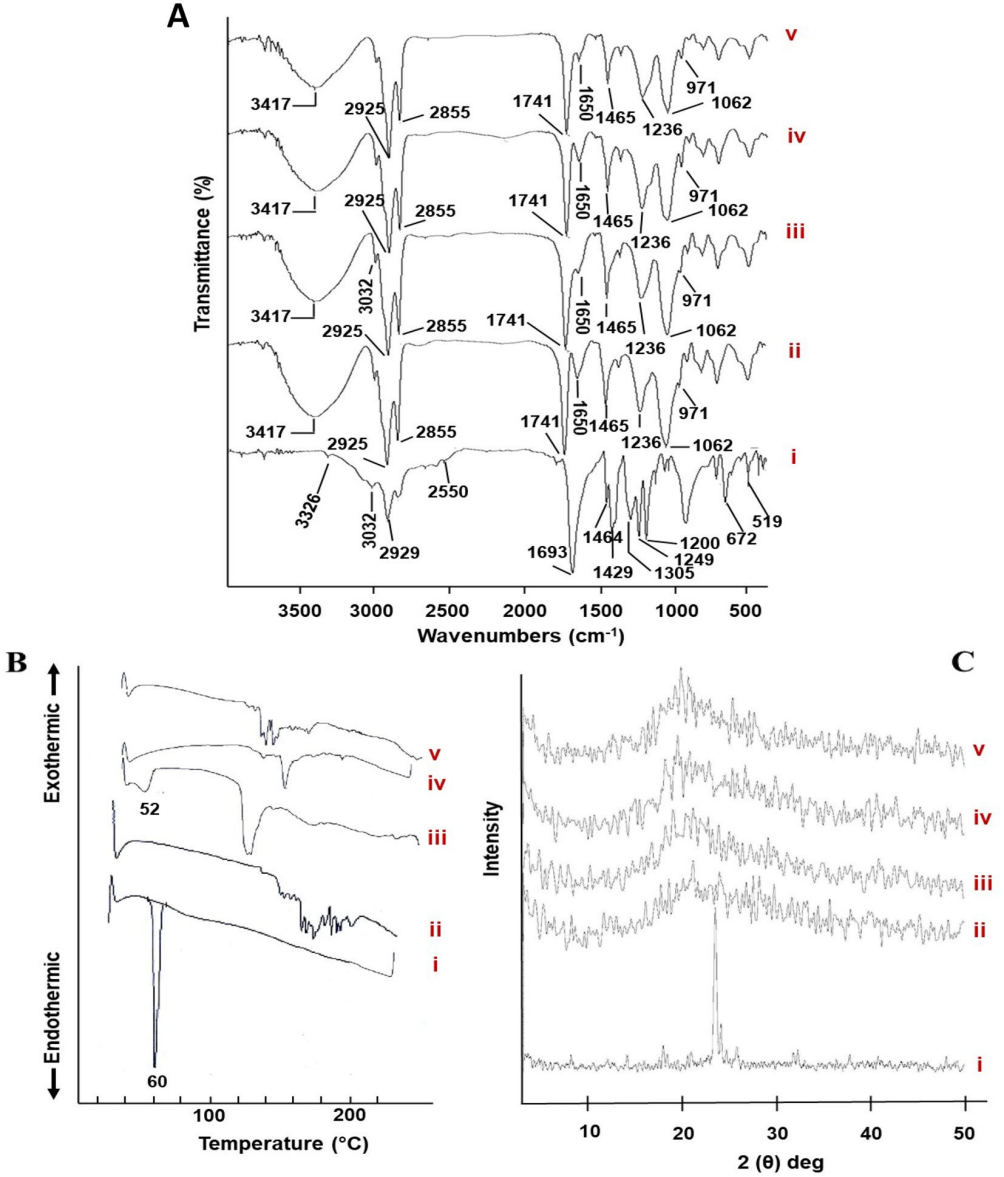
Solid characterization. (**A**) FT-IR spectra, (**B**) DSC thermograms, and (**C**) XRD patterns of pure ALA (i), SL (ii), physical mixture of ALA and SL (iii), NLPs_free_ (iv) and ALAN(v).

As depicted in Fig. 3A, the FT-IR spectrum of ALA (i) shows distinct peaks in the region of 3400–2500 cm^−1^ attributed to symmetric and asymmetric stretching vibrations of both hydroxyl (–OH) and methylene (–CH_2_) groups. The absorption band at 1693 cm^−1^ is due to the stretching vibration of the carbonyl (C=O) group. Besides, the obvious bands appeared between 1470 and 1350 cm^−1^ are correlated to bending vibrations of both (–CH_2_) and (–C–O–H) groups. The peaks at 1305, 1249 and 1200 cm^−1^ are due to O–C (carboxylic acid) stretching vibrations, while bands at 672 and 519 cm^−1^ are attributed to C–S as well as S–S stretching vibrations, respectively. In the infrared spectrum of SL (ii), the broad distinctive band located at 3417 cm^−1^ could be attributed to the presence of water. The peaks at 2925 and 2855 cm^−1^ represent the symmetric and asymmetric stretching vibrations of (–CH_2_) groups of the alkyl chains. Moreover, the spectral bands at 1741 and 1650 cm^−1^ are related to ester carbonyl (C=O) group stretching vibrations, while that at 1465 cm^−1^ typifies scissoring vibrations of (–CH_2_) groups. Besides, the polar head groups vibrations are confirmed by the presence of three main infrared shoulders at 1236 cm^−1^ representing the asymmetric stretching of the phosphate (–PO_2_^−^) group, 1062 cm^−1^ representing the symmetric (–PO_2_^−^) group stretching which partially overlapped with that at 1054 cm^−1^ indicating the C–O–P–O–C stretching modes, as well as 971 cm^−1^ representing the antisymmetric stretching vibrations of (–N^+^–CH_3_). Physical mixture spectrum (iii) of ALAN components namely, SL and ALA, 50 and 10 mg, respectively, shows the bands of SL, whereas those of ALA are either overlapped with that of SL, disappeared or appeared with diminished intensities as the result of dilution factor. Likewise, NLPs_free_ (iv) as well as ALAN (v) spectra were coincided with that of the physical mixture.

Figure 3B reveals DSC thermograms of pure ALA, SL, their physical mixture, NLPs_free_ as well as ALAN. The DSC curve of ALA (i) has a single melting endotherm at 60 °C. The corresponding DSC spectrum of SL (ii) exhibits a merged peak between 140 and 210 °C. Physical mixture spectrum (iii) shows slight changes in terms of broadening and shifting in the temperature of the drug-excipient “ALA-SL” melt. Besides, the thermograms of both lyophilized NLPs_free_ (iv) and ALAN (v) experience endothermic transitions across the temperature range of 120–180 °C, with clear absence of ALA’s melting point in that of ALAN (v). Figure 3C illustrates XRD diffractograms of pure ALA, SL, their physical mixture, NLPs_free_ as well as ALAN. ALA (i) displays intense XRD peaks at 23.5°, 24.1°, and 25.6° (2θ). Contrariwise, the broad profile of SL (ii) was observed. The physical mixture (iii) shows a broad profile of SL with clear absence of ALA distinctive peaks due to its comparatively small amount. Furthermore, the X-ray diffraction patterns of ALAN (v) coincided with that of NLPs_free_ (iv), together with ALA's peaks disappearance.

In the present study, ALAN formulation exhibited neither sedimentation nor flocculation over a storage period of 3 months at the two different storage conditions. Table 1 compiles the measured numeric values of Z-average, PDI, ZP and the average drug retention (%) for ALAN formulation throughout the designated storage period at the different conditions. Applying ANOVA for statistical comparison with the freshly prepared ALAN batches, insignificant inequality was detected concerning the evaluation parameters throughout the appointed storage period at long-term conditions (4 ± 1 °C). Contrarily, upon storage at accelerated conditions (25 ± 1 °C, 60 ± 5% RH), only significant (*p* < 0.05) increase in Z-average was elucidated, while the other evaluated parameters showed insignificant variations.

**Table 1:** The values of Z, PDI, ZP averages and drug retention (%) of ALAN aqueous dispersions stored at long-term storage conditions (4 ± 1 °C) and accelerated storage conditions (25 ± 1 °C, 60 ± 5% RH).

Evaluation parameters	Storage time		
	Zero time	1 month	3 months
**Long-term conditions (4 ± 1 °C)**			
Z-average (nm)	171.8 ± 2.60	164.23 ± 4.25	171.23 ± 2.29
Polydispersity index (PDI)	0.264 ± 0.01	0.267 ± 0.02	0.255 ± 0.01
Zeta potential (ZP, mV)	− 43.40 ± 3.35	− 41.77 ± 1.96	− 38.10 ± 0.98
Average drug retention (%)	100.00 ± 0.0	99.95 ± 0.20	99.91 ± 0.50
**Accelerated conditions (25 ± 1 °C, 60 ± 5% RH)**			
Z-average (nm)	171.80 ± 2.60	223.33 ± 2.57*	215.13 ± 1.10*^#^
Polydispersity index (PDI)	0.264 ± 0.01	0.281 ± 0.05	0.254 ± 0.01
Zeta potential (ZP, mV)	− 43.40 ± 3.35	− 38.13 ± 2.18	− 38.17 ± 1.33
Average drug retention (%)	100.00 ± 0.00	100.08 ± 0.12	99.82 ± 0.37

Each value represents the mean ± standard deviation (SD) (n = 3).

*Significant at *p* < 0.05 monthly vs. initial.

^#^Significant at *p* < 0.05 refrigeration vs. ambient conditions after 3 months.

### Effect of supplementing Tris-extender with ALAN on sperm characteristics in equilibrated buffalo bull semen (at 5 °C for 4 h)

As outlined in Table 2, the effect of ALAN on the sperm functionality at 5°C for 4 h was performed. The progressive motility, vitality and membrane integrity significantly improved in all ALAN groups (except ALAN25 for membrane integrity). Membrane integrity did not change significantly in the presence of ALAN (25 µg) as compared with the control group at 5°C for 4 h. Moreover, the ALAN50 had higher positive impacts on sperm abnormalities than other treated and control groups. High levels of ALAN (75 and 150 µg) decreased the sperm abnormalities without significance when compared with the control one.

**Table 2:** Effect of supplementing Tris-extender with alpha-lipoic acid loaded liposomes on sperm characteristics (%; means ± SE) in equilibrated buffalo bull semen (at 5 °C for 4 h).

Treatment^1^	Sperm characteristics (%)^2^			
	Progressive motility	Vitality	Membrane Integrity	Abnormality
ALAN0	71.0 ± 1.00^b^	73.6 ± 0.87^b^	74.0 ± 0.55^b^	11.0 ± 0.63^a^
ALAN25	77.0 ± 1.22^a^	79.4 ± 1.12^a^	78.6 ± 1.75^ab^	9.0 ± 0.45^b^
ALAN50	81.0 ± 1.87^a^	83.2 ± 1.46^a^	82.4 ± 1.96^a^	7.6 ± 0.24^c^
ALAN75	80.0 ± 1.58^a^	81.0 ± 1.58^a^	80.6 ± 1.83^a^	9.8 ± 0.37^ab^
ALAN150	81.0 ± 1.87^a^	82.8 ± 1.62^a^	82.8 ± 1.43^a^	9.6 ± 0.51^ab^

^1^Tris extender supplemented with different levels of alpha-lipoic acid loaded liposomes (ALAN) containing 0 (ALAN0), 25 (ALAN25), 50 (ALAN50), 75 ALAN75 and 150 ALAN150 (0, 25, 50, 75 and 150 µg/mL), respectively.

^2^a–c means denoted within the same column with different superscripts are significantly different at *P* < 0.05.

### Effect of supplementing Tris-extender with ALAN on sperm characteristics (%) in post-thawed buffalo bull semen (at 37 °C for 30 s)

The effect of ALAN on sperm characteristics (%) in post-thawed buffalo bull semen (at 37 °C for 30 s) are presented in Table 3. We observed that supplementing tris-extender with ALAN significantly improved progressive motility and vitality and had no effects on the sperm abnormalities and membrane and acrosome integrities of post-thawed buffalo semen thawed at 37 °C for 30 s. ALAN150 groups exhibited the best values of progressive motility and vitality compared with those in other groups. No significant differences for progressive motility in ALAN50, ALAN25 and the control groups were detected.

**Table 3:** Effect of supplementing Tris-extender with alpha-lipoic acid loaded liposomes (ALAN) on sperm characteristics (%) in post-thawed buffalo bull semen (at 37 °C for 30 s).

Treatment^1^			Sperm characteristics (%)^2^			
	Progressive motility	Vitality		Membrane Integrity	Acrosome Integrity	Abnormality
ALAN0	36.0 ± 1.87^c^	39.4 ± 2.75^c^		40.6 ± 2.40	90.6 ± 0.93	12.6 ± 0.93
ALAN25	40.0 ± 1.58^bc^	43.6 ± 1.94^abc^		43.0 ± 1.64	91.8 ± 0.86	11.6 ± 0.51
ALAN50	40.0 ± 1.58^bc^	42.2 ± 2.56^bc^		42.6 ± 3.19	92.6 ± 1.66	10.6 ± 0.68
ALAN75	45.0 ± 1.58^ab^	47.2 ± 1.59^ab^		48.0 ± 2.66	91.6 ± 1.03	10.6 ± 1.03
ALAN150	47.0 ± 2.00^a^	50.4 ± 2.56^a^		48.4 ± 2.38	91.0 ± 1.00	12.8 ± 0.97

^1^Tris extender supplemented with different levels of alpha-lipoic acid loaded liposomes (ALAN) containing 0 (ALAN0), 25 (ALAN25), 50 (ALAN50), 75 ALAN75 and 150 ALAN150 (0, 25, 50, 75 and 150 µg/mL), respectively.

^2^a–c means denoted within the same column with different superscripts are significantly different at *P* < 0.05.

### Effect of supplementing Tris-extender with ALAN on sperm characteristics (%) in post-thawed buffalo bull semen after incubation (at 37 °C and 5% CO_2_ for 2 h)

For insight to assess the potentiality long impacts of ALAN on semen parameters, we analyzed the semen characteristics after 2 h of incubation at 37 °C and 5% CO_2_ (Table 4). Significant changes in vitality and progressive motility of buffalo sperm as response to ALAN supplementation to extender were noticed. ALAN150 exhibited the best values of all studied sperm function (except abnormalities) in related to those in other groups. In most cases, no significant difference was found between control and ALAN25 group. The intermediate values of progressive motility, vitality and membrane integrity in buffalo sperm were found in ALAN25, ALAN50 and ALAN75 groups. The sperm abnormalities did not significantly influence by ALAN supplementation. ALAN provided better protection for vitality, membrane integrity and progressive motility in buffalo semen post-thawed incubated for 2 h at 37 °C and 5% CO_2_.

**Table 4:** Effect of supplementing Tris-extender with alpha-lipoic acid loaded liposomes (ALAN) on sperm characteristics (%) in post-thawed buffalo bull semen after incubation (at 37 °C and 5% CO_2_ for 2 h).

Treatment^1^	Sperm characteristics (%)^2^			
	Progressive motility	Vitality	Membrane integrity	Abnormality
ALAN0	30.0 ± 2.24^c^	33.6 ± 2.34^c^	34.4 ± 2.25^c^	13.6 ± 0.93
ALAN25	35.0 ± 1.58^bc^	38.4 ± 2.11^bc^	37.6 ± 1.21^bc^	12.0 ± 0.55
ALAN50	36.0 ± 1.87^b^	39.8 ± 1.50^ab^	39.0 ± 1.45^abc^	12.8 ± 0.58
ALAN75	39.0 ± 1.00^ab^	41.4 ± 1.57^ab^	40.6 ± 1.50^ab^	12.8 ± 0.97
ALAN150	43.0 ± 2.00^a^	45.4 ± 1.75^a^	43.0 ± 1.76^a^	14.0 ± 0.84

^1^Tris extender supplemented with different levels of alpha-lipoic acid loaded liposomes (ALAN) containing 0 (ALAN0), 25 (ALAN25), 50 (ALAN50), 75 ALAN75 and 150 ALAN150 (0, 25, 50, 75 and 150 µg/mL), respectively.

^2^a–c means denoted within the same column with different superscripts are significantly different at *P* < 0.05.

### Effect of supplementing Tris-extender with ALAN on antioxidant, oxidative markers enzymatic activities in extender of post-thawed buffalo bull semen

Data pertaining the impacts of ALAN on antioxidant, enzymatic activities and lipid peroxidation after thawing of cryopreserved semen of buffalo are illustrated in Table 5. Supplementation of ALAN at levels 75 and 150 µg/mL significantly improved the TAC, GR and catalase, with the best results. however, Results indicated that ALAN supplementation significantly increased all antioxidative response. The lowest values of LP were found in ALAN150 group followed by ALAN75 group, while the higher lipid peroxidation was detected in control one. All ALAN treatments significantly decreased AST and TAP values in seminal plasma. No significant differ was found among all experimental groups for ALT and ALP. Overall, supplementing of ALAN improved significantly TAC, CAT and GR, while significantly decreased MDA, AST, TAP and had no effects on ALT and ALP.

**Table 5:** Effect of supplementing Tris-extender with alpha-lipoic acid loaded liposomes (ALAN) on antioxidant, oxidative markers and enzymatic activities in extender of post-thawed buffalo bull semen (means ± SE).

Items^1^	Treatments^2^				
	ALAN0	ALAN25	ALAN50	ALAN75	ALAN150
**Antioxidative marks**					
TAC (mM/L)	0.17 ± 0.02^c^	0.19 ± 0.01^bc^	0.21 ± 0.01^b^	0.26 ± 0.01^a^	0.28 ± 0.01^a^
CAT (U/L)	254.2 ± 4.21^c^	283.4 ± 12.80^b^	343.7 ± 10.04^a^	353.8 ± 8.71^a^	370.7 ± 7.68^a^
GR (U/L)	110.6 ± 3.20^c^	129.3 ± 4.79^b^	129.6 ± 4.87^b^	143.2 ± 3.07^a^	152.6 ± 2.75^a^
**Lipid peroxidation**					
MDA (nmol/mL)	19.62 ± 0.22^a^	17.15 ± 0.17^b^	16.94 ± 0.20^b^	14.88 ± 0.35^c^	14.12 ± 0.21^d^
**Enzymatic activities**					
AST (U/mL)	51.6 ± 3.19^a^	44.4 ± 1.54^b^	44.4 ± 1.17^b^	44.0 ± 0.89^b^	44.8 ± 1.24^b^
ALT (U/mL)	12.4 ± 1.03	9.6 ± 0.60	10.4 ± 0.60	10.6 ± 0.93	10.8 ± 0.92
ALP (IU/L)	149.9 ± 7.05	151.8 ± 6.88	136.2 ± 9.38	131.8 ± 10.82	131.0 ± 7.98
TAP (U/L)	111.5 ± 2.71^a^	99.2 ± 2.21^b^	97.3 ± 2.17^b^	101.6 ± 4.30^b^	86.0 ± 3.26^c^

^1^TAC, Total antioxidant capacity; CAT, Catalase; GR, Glutathione reductase; MDA, Malondialdehyde; AST, Aspartate aminotransferase; ALT, Alanine aminotransferase; ALP, alkaline phosphatase; and TAP, Total Acid Phosphatase.

^2^Tris extender supplemented with different levels of alpha-lipoic acid loaded liposomes (ALAN) containing 0 (ALAN0), 25 (ALAN25), 50 (ALAN50), 75 ALAN75 and 150 ALAN150 (0, 25, 50, 75 and 150 µg/mL), respectively. ^a–d^means denoted within the same row with different superscripts are significantly different at *P* < 0.05.

### Effect of supplementing Tris-extender with ALAN on apoptosis-like changes of post-thawed buffalo bull sperm

As depicted in Table 6, the effects of ALAN on apoptosis-like changes of buffalo spermatozoa after thawing. The greater values of viable spermatozoa were found in semen samples supplemented with 150 µg/mL. Both ALAN50 and ALAN150 significantly decreased the percentages of early apoptotic spermatozoa compared with those in the other groups. There were no significant differ between ALAN25 and the control in relation to the early apoptotic spermatozoa. The apoptotic spermatozoa were significantly reduced in ALAN supplemented frozen-thawed groups compared to that in the control group. No significant difference was found with control in terms of the necrotic spermatozoa when compared with the supplemented groups.

**Table 6:** Effect of supplementing Tris-extender with alpha-lipoic acid loaded liposomes (ALAN) on apoptosis-like changes of post-thawed buffalo bull sperm (Annexin V/PI assay).

Treatment^1^	Sperm characteristics (%)^2^			
	Viable (A−/PI−)	Early apoptotic (A+/PI−)	Apoptotic (A+/PI +)	Necrotic (A−/PI+)
ALAN0	32.6 ± 0.09^e^	1.0 ± 0.09^b^	46.4 ± 2.74^a^	20.2 ± 2.92
ALAN25	40.8 ± 0.38^d^	3.0 ± 0.12^a^	37.6 ± 1.01^b^	18.7 ± 0.75
ALAN50	52.6 ± 0.49^c^	0.4 ± 0.03^c^	29.1 ± 0.03^c^	18.1 ± 0.49
ALAN75	56.2 ± 0.86^b^	1.1 ± 0.21^b^	22.8 ± 1.23^d^	19.9 ± 0.78
ALAN150	64.9 ± 0.20^a^	0.3 ± 0.06^c^	14.5 ± 0.06^e^	20.4 ± 0.20

^1^Tris extender supplemented with different levels of alpha-lipoic acid loaded liposomes (ALAN) containing 0 (ALAN0), 25 (ALAN25), 50 (ALAN50), 75 ALAN75 and 150 ALAN150 (0, 25, 50, 75 and 150 µg/mL), respectively.

^2^a–e means denoted within the same column with different superscripts are significantly different at *P* < 0.05.

### Sperm ultrastructure

**Figure 4:**
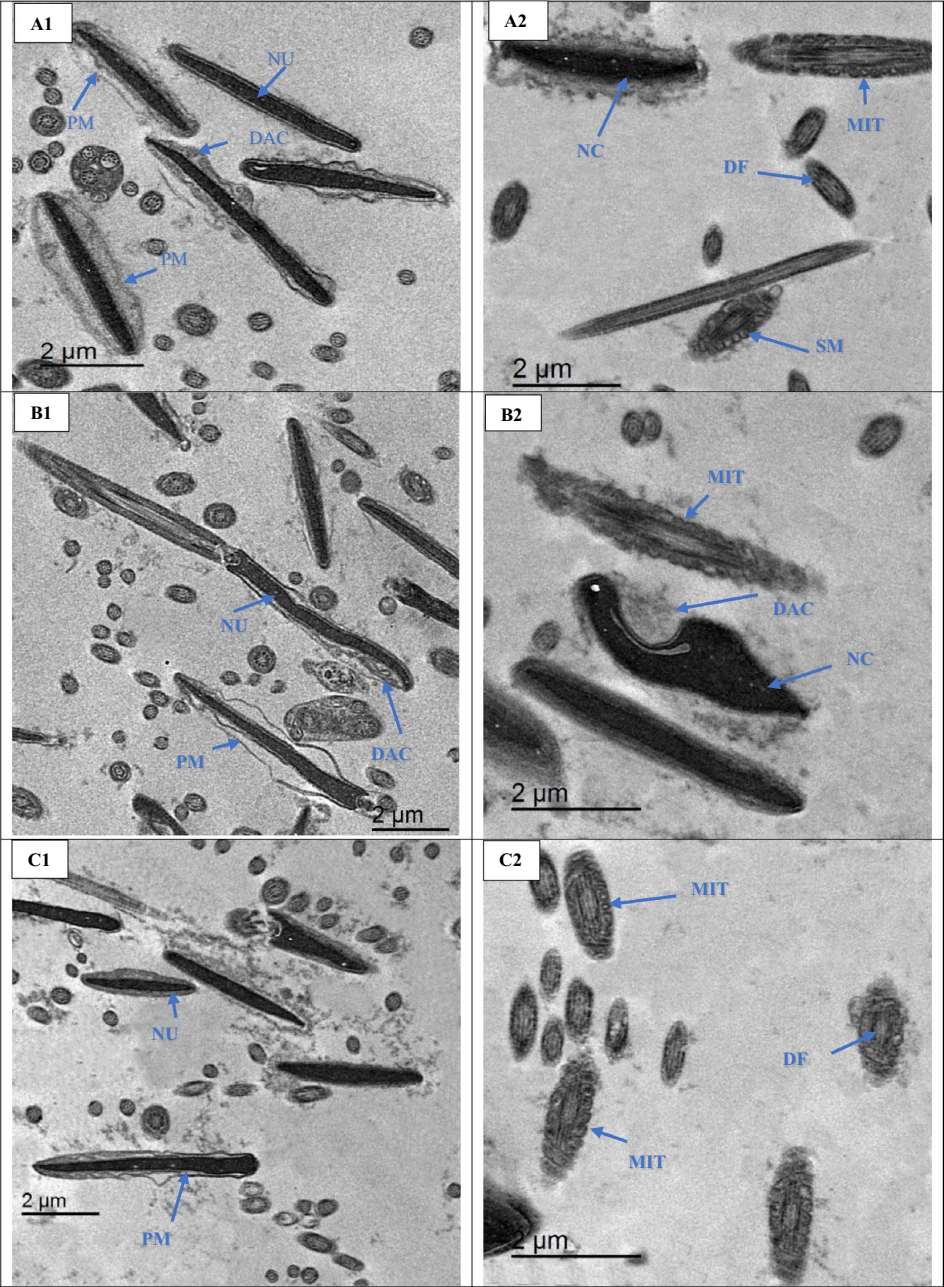

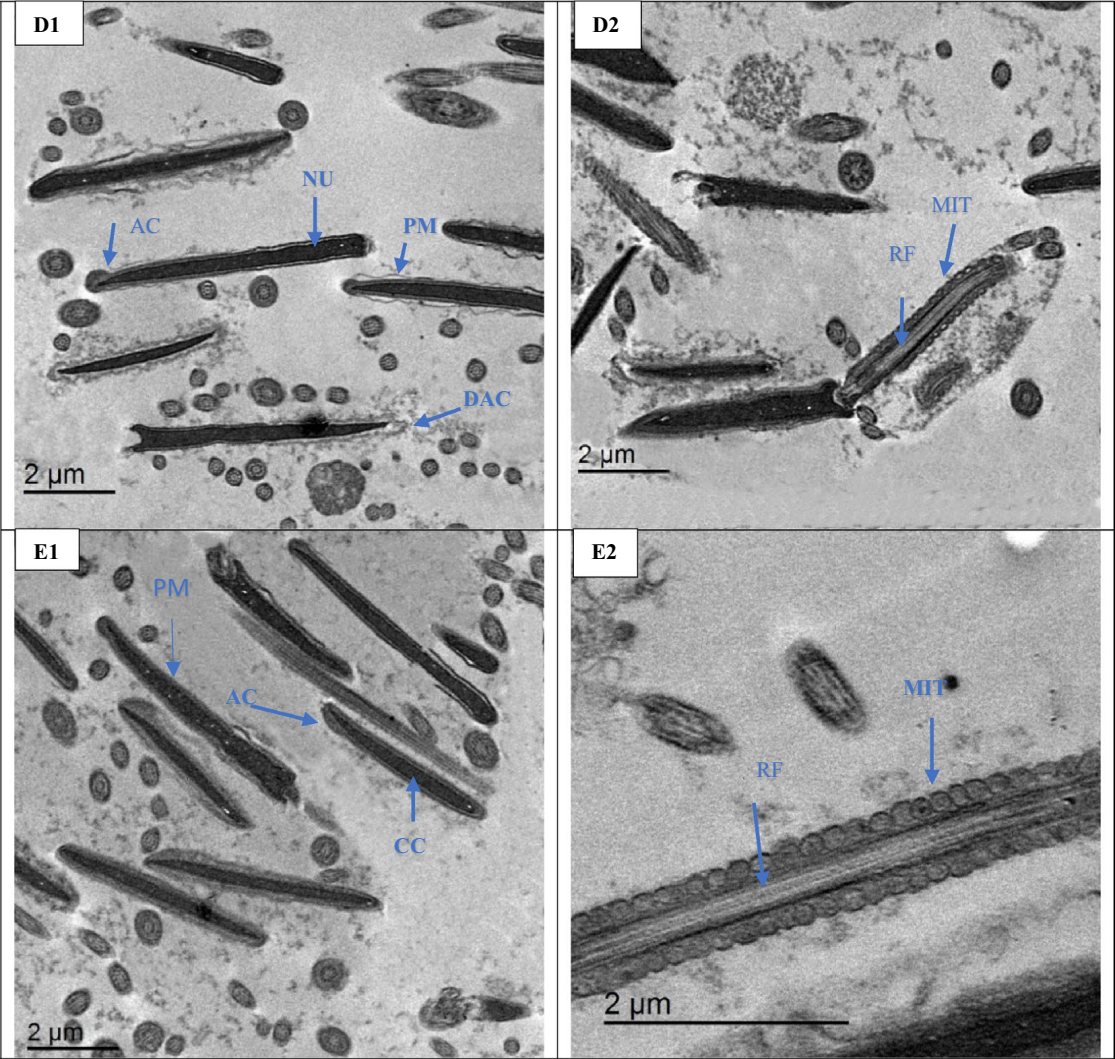
Transmission electron microscopy (TEM) micrographs of post–thawed sperm of buffalo in groups treated with 0, 25, 50, 75 or 150 µg/mL alpha-lipoic acid loaded liposomes (ALAN) (A1,2; B1,2; C1,2; D1,2 and E1,2 respectively). A1, A2) The control group (A1 for sperm head and A2 for sperm tail) showing nucleus (NU) without plasma membrane (PM), swollen plasma membrane, damaged acrosomal (DAC), necrotic chromatin (NC) and mitochondria (MIT) with distorted cristae. B1, B2, C1 and C2) The groups 25 and 50 µg ALAN supplemented with freezing extender, buffalo head sperm has moderate condensed chromatin, acrosomal swelling and some alterations in plasma integrities. Moreover, the same groups showing slight abnormal mitochondrial (MIT). D1, D2, E1 and E2) The groups 75 and 150 µg ALAN supplemented with freezing extender, buffalo sperm showing preserve plasma membrane structure (PM), condensed chromatin (CC), and regular flagellum (RF) with sustain of cristae membrane in MIT. CC; condensed chromatin, SM; swollen mitochondria, NC; necrotic chromatin, PM; plasma membrane, MIT; mitochondria, AC; intact acrosomal, DAC; Damaged acrosomal, DF; Disarranged of flagellum, RF; Regular flagellum.

As depicted in Fig. 4, plasma membrane of post-thawed of buffalo sperm was swelling, undulation and some degrees of damages, necrotic chromatin, and mitochondria (MIT) with distorted cristae in the ALAN0 group (Fig. 4A1, A2). While the ALAN25 and ALAN 50 groups exhibited moderate changes in the plasma membrane, moderate condensed chromatin, acrosomal swelling and some alterations in plasma integrities and slight abnormal mitochondrial (Fig. 4, B1, B2, C1 and C2). The plasma membrane structure, condensed chromatin, and regular flagellum with sustain of cristae membrane in mitochondria were observed in the ALAN75 and ALAN150 groups (Fig. 4, D1, D2, E1 and E2). The addition of ALAN at 75 or 150 µg/mL to the freezing extender of buffalo semen could protect the sperm form the damages induced by cryopreservation process.

## Discussion

Our outcomes exhibited that supplementation of freezing medium with ALAN maintains sperm viability, motility, and membrane integrity, this goal is plausible accomplished by enhancing the antioxidant capacity and reducing the lipid peroxidation. Decrease in lipid peroxidation and apoptotic like change in spermatozoa protects genome from DNA damage which may lead to enhance cryo-preservability of buffalo semen. Buffalo spermatozoa is very susceptible to cryo-damage induced by cryopreservation, due its high contents of fatty acids in their membrane structures^16,23^. Hence, antioxidants supplemented to freezing media could enhance sperm cryo-tolerance, and act as a potentiality strategy to reduce the adverse influences of oxidative stress triggered by cryopreservation^6,29–31^**.** With the advancement and quickly progress of nanomedicine sciences, the development of nano-encapsulated agent might introduce an effective, stable and more specific tool in improving semen cryopreservation^5,6,14,30,31^.

ALA, being hydro and lipo-soluble, was victoriously entrapped in the prepared NLPs which can entrap both hydrophobic and hydrophilic compounds^32,33^. Ultrafiltration of the resultant yellow translucent colloidal dispersion, to distinguish between entrapped ALA inside NLPs and the unentrapped one that was outside, was performed as recently reported^34^. Considering the following equation of a spherical particle “Eq. (2)”, using M of ALA as 206.33 Dalton (Da), confirms the ability of the used Ultra-4 Centrifugal Filter Units to allow only the passage of unentrapped ALA whereas entrapped ALA can’t.

2 $$\rm{R_{min} = 0.066 M^{1/3}}$$

Where, R_min_ is the minimal radius of a sphere in nanometer (nm) that could contain a given mass of M in Da^35^. Hence, unentrapped ALA in the ultrafiltrate could be determined spectrophotometrically at 242 nm without further dilution. The attained high EE% of ALA was analogous to that obtained when^36^ encapsulated ALA in NLPs that would supplement the recommended levels of such biomolecule “ALA” effectively and conveniently. Such data suggested that NLPs are suitable for ALA encapsulation with high EE % which is an insistent demand to reduce both together the utilized carrier materials as well as the manufacturing cost, while fewer nano-formulation of medication will be used to deliver the required therapeutic dosage^37^.

Concerning nanoparticulate systems, not only their Z-average values but also PDI ones expressing the breadth of the particle size distribution are characters which have a prime influence on biodistribution, bioavailability besides colloidal dosage form stability upon storage. It can be deduced that the attainable small Z-average of ALAN might render them able to penetrate sites such as the plasmatic membrane of spermatozoa. Similarly, small PDI values showed narrow distribution, homogeneously prepared formulations, and enhanced colloidal stability without formation of aggregates^38^. The ALAN spherical morphology, with little or no aggregation, could be attributed to the high negative ZP value. Moreover, enhanced stability of the NLPs formulations under physiological conditions might be speculated^38^.

Regarding the FT-IR spectra, the observed peaks of ALA clearly describe its chemical nature^18,39,40^**.** Besides, analogous peaks were previously reported for SL^41,42^**.** ALAN spectrum indicates ALA entrapment in the prepared NLPs. As a powerful analytical tool, DSC can elucidate thermodynamic differences related to morphological changes during NLPs formulation. Pure ALA thermogram indicates its crystalline nature^43^, while similar one for SL was documented earlier^44^. Slight changes in the melting endotherm of both drug and lipid physical mixture might not necessarily indicate potential incompatibility, where physical mixing is known to influence the purity of each component with subsequent changes in the peak shape and enthalpy^45^. The ostensible absence of ALA melting point in ALAN' spectrum confirms its encapsulation and suggests its conversion to an amorphous or molecular dispersion during the run. Besides, the endothermic transitions of both NLPs_free_ and ALAN seem likely for SL, where it was reported that the phase transition temperature of colloidal dispersion was always much lower than that of the pure anhydrous lipid^38,45,46^. As for the XRD diffraction patterns**,** the intense peaks of ALA confirm its crystalline nature^47^, whilst the amorphous nature of SL was indicated. Moreover, ALAN diffractogram denotes ALA's encapsulation within liposomal matrix in amorphous or molecular dispersed state. Analogous findings were previously reported^48^. Generally, shelf-life stability of NLPs is determined by measuring the crucial parameters namely; physical appearance, Z-average, PDI, ZP besides average drug retention (%), which determine their physical and chemical stabilities^21^. The measured numeric data highlighted the stability of ALAN at 4 ± 1 °C for 3 months expressed by uniform nanosize range along with homogenic dispersion.

The assessment of progressive motility might provide the first vigorous markers of fertilizing capacity and sperm freezability. In our data, sperm progressive motility at equilibrated time at 5 °C for 4 h or freeze–thaw at 37 °C for 30 s or incubated after thawing at 37 °C for 2 h was improved significantly (*P* < 0.05) in all exogenous supplemented ALAN groups as compared to the control one. ALAN150 group exhibited the best values for sperm motility when compared with the other groups. In line with or results, Najafi et al.^49^ found that exogenous supplementation of ALA loaded nano-lipid carrier (30 µM) to the freezing media improved significantly total and progressive motility of rooster sperm as compared with the control. Moreover, the addition of ALA to freezing extender enhancing (*P* < 0.05) sperm motility in boar^12^, ram^11,17^, buffalo^29^ and goats^3,15^. In other study on human, adding ALA (0.02 mM) to sperm preparation media can pointedly sustain sperm motility and viability from oxidative damages persuaded through centrifugation process^50^. However, previous study of Gohar et al.^29^ discovered the effects of ALA on buffalo semen freezability, there were no previous reports on the potentiality of using ALAN as a cryoprotective agent on freezing extender in buffalo sperm cryopreservation process. ALA plays an important role in energy production and metabolism by acting as a mitochondrial coenzyme. This feature leads enhance the availability of mitochondrial coenzymes, hence increases the fortification alongside with oxidative stress subsequently decreases the incidence of mitochondrial dysfunction, ultimately warranting suitable ATP for sperm motility^49^. It could be said that this improvement of sperm motility indicated in the current research might be owing to the new form of ALA especially ALAN, which introduce a significant improvement in the water availability, more stability and effectively to target the mitochondria function and thereafter enhance sperm functionality. ALA’s role in the Krebs cycle may aid in the production of ATP, and thus it may increase spermatozoa motility and help in fusion with the ovum. Moreover, ALAN generates a vigorous shield and protects membrane form ROS damage and thus preserves sperm motility and viability.

Semen processing, mainly semen handling, dilution and deep-freezing decreases sperm natural antioxidant in sperm which finally make them susceptible to the ROS produced and thus weakening plasma and acrosome membranes, viability and DNA integrity of spermatozoa. A functional membrane is very much crucial for sperm motility, and several other physiological events including capacitation, acrosome reaction, and fertilization^15^. Studies have shown that the functional integrity of plasma membrane was comprised by cryopreservation process^1,3,7,26^. Plasma membranes overhead and mid-piece are more prone to oxidative damage as significant distortion in these regions was observed after cryopreservation of bull spermatozoa^21,23^. We found that the addition of ALAN (50–150 µg/mL) provided a significant protective effect for plasma membrane integrity in equilibrated buffalo bull semen (at 5 °C for 4 h) and in post-thawed buffalo bull semen after incubation (at 37 °C and 5% CO_2_ for 2 h). Similar with our results, addition of ALA (0.5 and 1 mmol/L) improved significantly the membrane integrity of ram sperm after cryopreservation process^17^. Additionally, the plasma membrane integrities of post-thaw boar sperm were significantly enhanced by adding ALA to the extender^12^. Our data display that ALAN50, ALAN100 and ALAN150 provided better protective effect for membrane integrity of buffalo sperm after two hours of incubation, however there were no significant effect on abnormalities, membrane and acrosome integrities in post-thawed buffalo bull semen (at 37 °C for 30 s). Furthermore, Addition of ALAN might have restrained the membrane related to oxidative injury and enhanced the membrane intactness of buffalo spermatozoa. As compared with our results, there were no previous studies have been used the ALA (especially nano form) applying in buffalo cryopreserved semen. So, it is difficult to compare with other studies, however, we tried to be mentioned only the incorporation of ALA in freezing extender of different animal species. The protective effect of ALAN for sperm membrane integrity could be attributed to the higher antioxidant activity of ALA^29,49^. This result shows that addition of ALA can maintain membrane function and DNA integrity by reducing oxidative damage.

Extreme synthesis of ROS induced by cryopreservation process leads to induction of oxidative stress, lipid peroxidation, DNA damage and decrease the antioxidant defense system of sperm^12,51^. ALA, being a mitochondria-targeted antioxidant, diminishes ROS synthesis, which is principally accountable for lipid peroxidation. Consequently, comparatively low MDA levels were detected in all ALAN- supplemented groups at the post-thaw phase in seminal plasma of buffalo semen. Significantly higher antioxidant markers including TAC, CAT, and GR were noticed in ALAN supplemented groups than the control group, which might be because of targeted enhancing the antioxidant defense. The activities of AST and TAP were decreased significantly by the ALAN supplementation. The reduction of TAC or CAT in the seminal plasma of cryopreserved semen might be attributed to the dilution and cryopreservation process and these are tired in counteracting extreme ROS^52^. Several studies have demonstrated that supplementation of some nanomaterial such nanocurcumin^5,31^, and ellagic acid loaded liposomes^53^ and alpha-lipoic acid–loaded nanostructured^49^ into the freezing media could enhance the antioxidant capacity of seminal plasma in rabbit and rooster, respectively. In the present research, ALAN was effectively protected spermatozoa form the adverse effects of ROS via enhancing the defense system. The enzymatic system in the seminal fluid is evidenced to be vital for numerous metabolic events in spermatozoa, which provide energy for sperm functionality and fertilization^51^. Our research indicated that the activities of AST and TAP were significantly (*P* < 0.05) reduced in the supplemented ALAN groups as compared with the control.

Apoptosis-like changes are important for phosphatidyl serine externalization in plasma membranes of spermatozoa^54^. It has been reported that the freezing process promotes the incidence of apoptotic spermatozoa and thus reduce the fertility capacity of cryopreserved semen^5,55^. In this research, we reported that ALAN supplementation in the freezing media resulted in a significant reduction apoptosis-like changes (apoptotic and early apoptotic spermatozoa) of buffalo sperm when compared with the control value. In line with our data, previous reports have exhibited the percentages of apoptosis-like changes were significantly reduced by natural antioxidant; selenium nanoparticles^6^, zinc nanoparticles^30^, and nano-phytochemical^5,31^ supplemented to the freezing media. However, the use of encapsulated ALA in the liposomes is a new strategy applied in the field of semen cryopreservation. Authors suggested that this protective effect of ALAN might be attributed to the smaller size of nanoliposomes which penetrating the mitochondrial membranes and protect them for oxidative stress produced in it. Previous report indicated that nanoliposome enhance fatty oxidation in mitochondrial, thereafter provide the energy for sperm which explaining the improvement of sperm motility and enhance the defense system^14^.

Cryopreservation process triggered several alterations in the plasma membrane, nucleus, acrosomes, mitochondria of spermatozoa^52,56^. Moreover, those alteration in sperm structure might resulted in apoptosis and necrosis like changes during cryo-storage and freezing–thawing process^1,2^. Our previous investigations indicated that the addition of some natural antioxidant (nanocurcumin, selenium nanoparticles and L-cancrinite) preserved plasma and acrosome membranes and protected the ultrastructure integrity of the cryopreserved spermatozoa in bull and rabbit bucks^4–6^.These findings revealed that the ALAN fortification to the freezing media of buffalo semen could present a substantial shield from cryo-damage by maintaining the integrity and functionality of the sperm membrane. Although our data are promising, further study is needed to explore the impacts of nanoliposomes as a lipid carrier with other antioxidant which are accountable for its favorable influences on cryopreservation of buffalo sperm.

## Conclusion

Our findings propose that addition with ALAN could provide superior cryoprotective capability for frozen–thawed buffalo spermatozoa. ALAN meaningfully enhanced motility, viability, plasma and acrosomal membrane integrities of buffalo spermatozoa. The addition of ALAN also enhanced the antioxidant ability, including TAC, CAT, and GR as well as reducing the releasing activities of AST, TAP and lipid peroxidation (MDA). The apoptotic like changes of cryopreserved buffalo sperm were significantly reduced as response to supplementation of ALAN to freezing media. Moreover, ALAN supplementation resulted in preserve the ultrastructure (plasma and acrosomal membranes, and nucleus) of cryopreserved buffalo spermatozoa. Further studies are required to elucidate the transcriptomic or proteomics changes in spermatozoa after supplemented of nanoliposomes in other mammalian species. This data could provide a new approach for developing a new cryoprotective agent during semen cryopreservation in buffalo.

## Data Availability

The authors declare that the data supporting the findings of this study are available within the paper.
